# The Utility of 3D Printing for Surgical Planning and Patient-Specific Implant Design in Maxillofacial Surgery: A Narrative Review

**DOI:** 10.7759/cureus.48242

**Published:** 2023-11-03

**Authors:** Tanvi S Singh, Nitin Bhola, Amit Reche

**Affiliations:** 1 Oral and Maxillofacial Surgery, Sharad Pawar Dental College and Hospital, Datta Meghe Institute of Higher Education and Research (DMIHER), Wardha, IND; 2 Public Health Dentistry, Sharad Pawar Dental College and Hospital, Datta Meghe Institute of Higher Education and Research (DMIHER), Wardha, IND

**Keywords:** patient-specific implant design, 3d printing, rapid prototyping, maxillofacial reconstructive surgery, additive manufacturing

## Abstract

Maxillofacial reconstructive implants are typically created in standard shapes and have a widespread application in head and neck surgery. During surgical procedures, the implant must be correctly bent according to the architecture of the particular bones. Bending takes practice, especially for untrained surgeons. Furthermore, repeated bending may increase internal stress, resulting in fatigue in vivo under masticatory loading and an array of consequences, including implant failure. There is a risk of fracture, screw loosening, and bone resorption. Resorption, infection, and displacement are usually associated with the use of premade alloplastic implants and autogenous grafts. Recent technological breakthroughs have led to the use of patient-specific implants (PSIs) developed by computer-designed additive manufacturing in reconstructive surgery. The use of computer-designed three-dimensional (3D)-printed PSI allows for more precise restoration of maxillofacial deformities, avoiding the common difficulties associated with premade implants and increasing patient satisfaction. Additive manufacturing is something that refers to a group of additive manufacturing methods. This technique has been quickly used in a variety of surgical procedures. The exponential expansion of this technology can be attributed to its enormous surgical value. Adding 3D printing to a medical practice can be a rewarding experience with stunning results.

## Introduction and background

Maxillofacial reconstruction is a popular medical procedure that has been extensively utilized in the elimination of tumors and the treatment of trauma. Surgical implants tend to be mass-produced in conventional formats, which must be manually bent during surgery to match the specific patient's bone structure. Shaping these plate-shaped implants takes a long time and is quite susceptible to errors for novice surgeons [1]. Furthermore, to attain the correct form, repeated bending is frequently required, resulting in internal compressive stress accumulation. Under in vivo occlusal forces, stressed implants have been shown to suffer from fatigue, causing plate fracture, corrosion, the loosening of screws, and bone resorption, among other issues. The usage of three-dimensional (3D)-printed patient-specific maxillofacial reconstructive implants has increased in recent years. The reduction of surgical process time and patient recovery time is critical in lowering healthcare provider and patient costs while simultaneously enhancing patient outcomes. Disruptive technologies such as additive manufacturing and digital 3D modelling have improved preoperative planning and therapy, resulting in the formation of a new field. Anatomical methods, surgical debridement guides, and designing models and prosthetics are examples of patient-specific assistive and implantable devices [2].

It was suggested in 1979 to use 3D medical imaging, to reconstruct a physical model. Subtractive manufacturing (milling) was available at the time, but rapid prototyping (RP) (3D printing) was not. The most popular method for developing an original part before the invention of 3D printers was to utilize a computer numerical control (CNC) machine. Typically, this method begins with a blank block of material that is gradually molded into the final portion. In the same year, the first anatomical model based on medical imaging was created [3]. Medical applications for 3D printing began to be seriously investigated in the late 1980s. The first 3D printing method, stereolithography (SLA), was created in 1994 and was the first to be implemented in the biomedical industry. Oral and maxillofacial surgery was the first branch to implement this technology. A multidisciplinary team is necessary to use 3D printing in the medical industry [4]. The majority of surgeons are unfamiliar with 3D printing methods, and most low-cost printers necessitate basic engineering skills for troubleshooting and computer-aided design (CAD). We will go through imaging modalities, the application of medical imaging for 3D modelling, 3D printing procedures, accessible materials, and the time and cost of 3D printing in a step-by-step manner [5].

## Review

Methodology

A comprehensive examination of the utility of 3D printing for surgical planning and patient-specific implant (PSI) design in maxillofacial surgery was performed. Multiple electronic databases, including PubMed, Medical Literature Analysis and Retrieval System Online (MEDLINE), and Google Scholar, were searched via the following keywords and combinations: "maxillofacial reconstructive implants," "3D printing," "additive manufacturing," "patient specific implant design," "3D printing processing and maxillofacial reconstructive surgery," and "patient specific implant and rapid prototyping." The search was performed in July 2023, which covered articles published between 2010 and 2023. Aside from exploring electronic databases, reference lists that included appropriate articles and review papers were manually evaluated in order to find new studies. Only articles that had been peer-reviewed and published were considered for inclusion. The eligibility of the titles, abstracts, and full-text publications was determined by two separate reviewers, with any disputes resolved through discussion and consensus. The wide literature search aimed to incorporate relevant publications and provide a comprehensive assessment of the relevance of 3D printing in maxillofacial surgery for surgical planning and patient-specific implant design.

Inclusion and Exclusion Criteria

**Figure 1:**
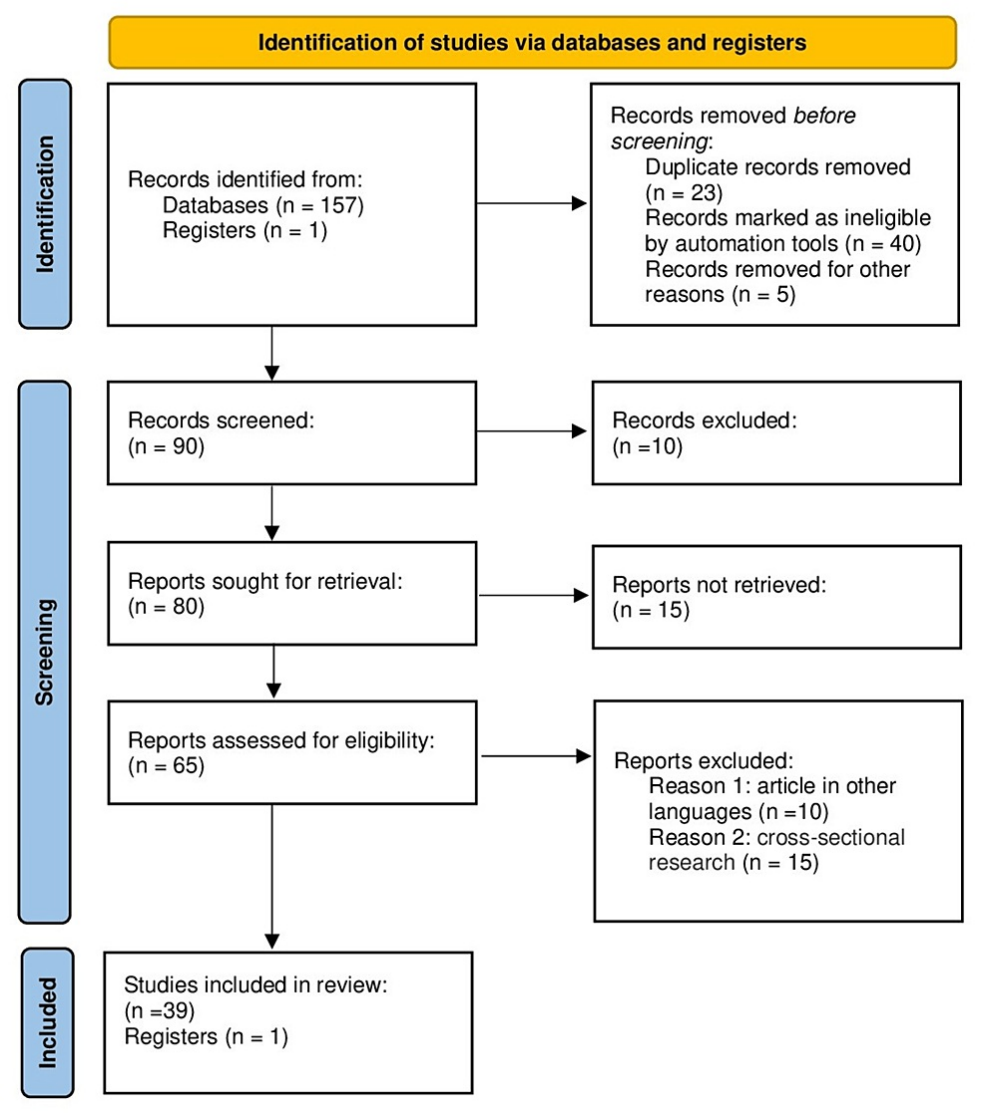
PRISMA flowchart of search strategy PRISMA: Preferred Reporting Items for Systematic Reviews and Meta-Analyses

The inclusion criteria comprised review articles, abstracts, summaries, and studies evaluating the usefulness of 3D printing for surgical planning and patient-specific implant design in maxillofacial surgery. Along with these studies published only in English, full-text articles and evaluations evaluating the practicality and acceptance of 3D printing in the contemporary era were also included. Cross-sectional research, prospective and retrospective investigations, and randomized controlled trials were among the exclusion criteria. In order to avoid translation bias, studies published in languages other than English were not included. Figure 1 shows the Preferred Reporting Items for Systematic Reviews and Meta-Analyses (PRISMA) flowchart for the literature search.

Materials for three-dimensional (3D) printing

Numerous degradable polymers, including polyglycolic acid (PGA), polylactic acid (PLA), and poly(lactic-co-glycolic acid) (PLGA), have been researched for maxillofacial reconstruction [6]. In vivo, solid PLGA has bone regeneration properties, and metabolic processes can eliminate the by-product [7]. Under mechanical stress, large PLGA prostheses exhibit a fast loss of strength and molecular weight, resulting in bulk disintegration. In a recent study, a 3D-printed polycaprolactone (PCL) scaffold with dual spatial and temporal growth factor administration was employed to evaluate the regeneration of the temporomandibular joint articular disc [8,9]. Although the scaffold's mechanical properties were similar to those of the native tissue, stress shielding poses a danger of nearby articular surface damage when a tougher polymer matrix is introduced into a soft cartilaginous tissue. In their study of the mechanical properties of cement for mandibular regeneration utilizing poly(propylene fumarate) (PPF) polymer, Lalwani et al. found that cross-linked microparticles significantly increased [10]. This composite's biocompatibility and cell attachment were both excellent. Another case study highlighted the potential for 3D-printed scaffolds in the craniofacial area to be turned into patient-specific applications [11]. Electron beam melting was used to make anatomically correct titanium mandibles. An inquiry into proof of concept looked into the potential use of 3D-printed ceramic-based implants for craniofacial reconstruction [12]. The personalized scaffolds were 3D-printed from a bio-ceramic powder bed, and the investigation culminated in the fabrication of a brushite/monetite resorbable implant. This has been studied further by embedding the framework in a 3D-printed human skull model and securing the bio-ceramic implant with titanium screws and plates [13]. The implant's structural feasibility was not tested, despite the fact that this is a compelling proof of concept; therefore, more research is necessary before using bio-ceramics in a load-bearing site [14].

Different Degradable and Non-biodegradable Materials Used for 3D Printing

Natural substances such as collagen, alginate, and chitosan are examples of degradable materials. These substances lack mechanical strength but have good biocompatibility and an extracellular matrix-like architecture [15]. Degradable synthetic materials (PCL and PLGA) have the highest possible mechanical strength, osteoconductive characteristics, and biocompatibility; nevertheless, their primary drawbacks are slow decomposition and the creation of a highly acidic environment [16].

Metals such as titanium, which exhibit high mechanical properties, biocompatibility, and corrosion resistance, are among the non-biodegradable materials utilized in 3D printing. However, titanium has the drawback of slowly releasing material particles. Ceramics (calcium phosphates) are another non-biodegradable substance; while they exhibit great osteoconductivity, they are fragile [17,18].

The process of 3D printing 

Rapid Prototyping

Rapid prototyping (RP), a CAD/computer-aided manufacturing (CAM) method, was primarily developed to manufacture commercial prototypes. It has made it easier for the industrial sector to create and produce huge quantities of precision parts in a timely and accurate manner. This technique has been used in the field of medicine for the previous 20 years with promising results [19]. The method comprises rapidly building prototypes or working models to aid in the creation and testing of various design aspects, ideas, concepts, functionality, and, in certain circumstances, outcome and performance.

**Figure 2:**
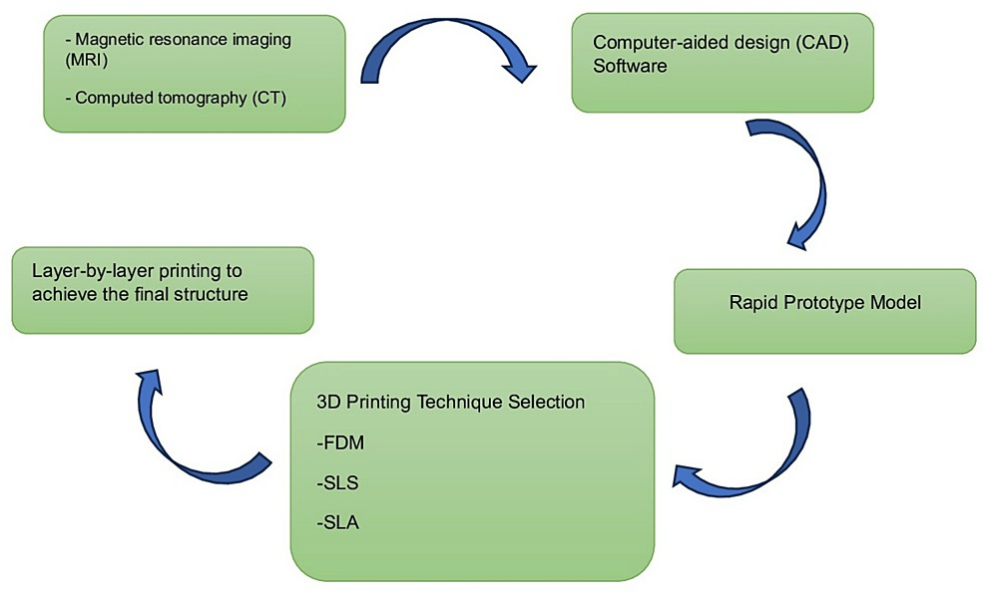
Flowchart of 3D printing process FDM, fused deposition modelling; SLS, selective laser sintering; SLA, stereolithography

Fast prototyping can be accomplished in three ways. Each has a distinct manufacturing technique. Fused deposition modelling (FDM), stereolithography (SLA or SL), and selective laser sintering (SLS) are three of them [20]. The material of choice is placed within the machine in a series of thin layers depending on the design data provided by the CAD program. Despite the fact that the underlying notions are the same, each technique operates slightly differently (Figure 2) [21].

Stereolithography

The most popular and widely used rapid-prototyping process is stereolithography. Stereolithography (SLA) is popular because it can produce high-accuracy, isotropic, and watertight prototypes and parts in a range of advanced materials with fine features and smooth surface finish [22]. It is an ideal solution for creating prototypes because it creates highly accurate, durable objects fairly quickly and relatively inexpensively. SLA machines can even create oddly shaped objects, which can be difficult to produce using traditional prototyping methods. SLA has undergone four generations of important technological innovation in the 40 years since its development in the 1980s. These advancements have resulted in a diverse spectrum of stereolithography systems with considerably enhanced resolution, throughput, and material choices enabling the creation of complex 3D structures and devices [23]. Stereolithography (SLA), the first kind of additive manufacturing, creates 3D things by selectively solidifying a liquid resin through a photopolymerization reaction. A vat containing liquid photopolymerized resin makes up the SLA. A laser positioned on top of the vat travels in 1 mm cross-sectional increments to match the slice intervals specified during the CT formatting procedure. On contact, the resin's surface layer is polymerized by the laser. The model's previously polymerized resin layer is carried away by a mechanical table that is just below the surface once the first slice is finished. The layer above the layer that was previously polymerized is now being polymerized by the laser. A comprehensive stereolithographic model may be produced in this way [24,25].

Stereolithographic Surgical Templates

A surgical template is the most common technique to communicate an implant placement plan to the patient, whether it was made on a cast or electronically on a computer, because a virtual plan developed from a cone-beam computed tomography (CBCT) scan can incorporate surgically relevant information while also embracing restorative goals. From a virtual blueprint, there are numerous techniques to build surgical templates. Rapid-prototyping SLA is an additive method for milling surgical templates [26]. Using the most popular approaches, the templates may be sterilized without losing any of their qualities. These include formaldehyde at 80°C and low-temperature steam. Stereocol resin was used to create the template itself. Both the practitioner and the patient benefit greatly from the use of stereolithographic templates. This innovative process saves a great amount of time [27]. No second-stage abutment connection surgery, impressions, or extra clinical or laboratory investigations are required. The amount of time needed is minimal, a single one-hour surgery as opposed to many, lengthier visits when using the conventional implant techniques.

Fused Deposition Modelling

A physical model is constructed using the non-laser technique of fused deposition modelling (FDM), which deposits layers of thermoplastic material one at a time. According to a study, anatomical models made via fused deposition modelling can be utilized as templates for the production of unique cranium implants, providing a technological advantage over standard cranioplasty approaches [28]. The restoration of symmetry in regions that are concerned with appearance can be facilitated by the mirror image of the nondeficit side [29]. In a comparison of models generated using the two methods, FDM and RP SLA models were found to have acceptable precision and strength; however, FDM was assessed to be more cost-effective. Similar dimensional accuracy is demonstrated by both models for bigger features [30]. The SLA provides an improved surface finish and more precision in tiny details. The FDM model has a lower cost of production than the SLA model.

Selective Laser Sintering

It is a free-form manufacturing technique that uses the thermal fusing (sintering) of powdered materials to create patterns. The STL files from 3D computer data are instantly translated into SLS models, which are then thinly sliced by the corresponding software. The laser sintering machine builds the sculptures on a movable platform by depositing thin layers of the pattern material [31,32].

Time Requirement

In the 3D printing process, processing 3D image data and/or manufacturing digital models with CAD can be time-demanding. This procedure is getting more user-friendly and automated as software progresses and workflow automation improves. It is worth mentioning that saving 10 minutes in the operating room equals conserving one hour of labor on a 3D-printed object [33]. More importantly, immediate patient benefits such as reduced anesthetic exposure, lower blood loss, and a better surgical outcome may be invaluable.

Cost

Because the majority of 3D printing systems are no longer patent-protected, their prices have lately dropped. It is not commonplace to use a professional 3D printing service, and it can save money, especially when printing in small quantities [34]. When printing in big quantities, purchasing a printing system is more cost-effective.

Pros of 3D printing

3D printing makes it possible to create and manufacture more complicated designs than traditional manufacturing techniques. When adopting 3D printing, design constraints that existed in more traditional techniques are simply no longer an issue [35]. Another advantage of print-on-demand is that, unlike traditional production methods, it does not necessitate a large amount of storage space. This saves both space and money because bulk printing is not required unless absolutely necessary. Changes to designs can be made for a low cost by modifying individual files rather than wasting money on tools and obsolete stocks. Although other metals may be used, plastic is the principal material for 3D printing. Plastics, on the other hand, have advantages because they are lighter than metals. This is critical in industries such as automotive and aviation, where weight reduction is important and can result in improved fuel economy. Additionally, pieces can be made from customized materials to offer certain qualities such as heat resistance, increased strength, or water repellency.

Unlike sculpted or machined components, 3D printing can create an object in a couple of hours, depending on the part's complexity and design. 3D printing can save time not just during the production phase but also during the design process, by providing ready-to-print STL or CAD files. Although 3D printing is a single-step production procedure, it saves both time and funds over using multiple pieces of equipment. When using 3D printers, operators do not have to be present at all times; they can be prepared and then facilitated to do the process [34]. Although owning 3D printing equipment is costly, you may be able to save money by outsourcing your project to a 3D printing service provider. 3D printers are becoming more inexpensive as more local service providers provide manufacturing outsourcing services. Particularly, compared with the increasingly traditional manufacturing procedures utilized by countries such as China, this saves time and eliminates the need for costly transportation costs. Because it decreases the quantity of material waste required, this procedure is environmentally benign. The environmental benefits increase when you include factors such as improved fuel economy from using lightweight 3D-printed parts [36]. 3D printing is being used in the medical profession to help save lives by creating human organs such as livers, kidneys, and hearts. Some of the most significant technology advances are being made in the healthcare field, where new applications and enhancements are constantly being produced [37].

Cons of 3D printing

Even though 3D printing can generate goods from a wide range of polymers and metals, the raw material availability is limited. The reason for this is due to the fact that not all metals and polymers can be heated to 3D printing temperatures. Moreover, only a small proportion of these printable materials are food-safe, and many of them are not recyclable. 3D printers' small print chambers now limit the size of the parts that may be generated. Something bigger will necessitate printing in multiple pieces, which will then be assembled after manufacturing. Since the printer needs to generate more pieces before engaging manual labor to join the parts, the price and manufacturing time for larger products may rise [38]. In contrast to more traditional procedures, such as injection molding, where large-volume production may be more cost-effective, 3D printing has a set cost [39]. While 3D printing has a lower initial investment than other manufacturing techniques, the cost per unit does not fall as much as injection molding when scaled up to mass-produced large volumes. By using additive manufacturing, commonly known as 3D printing, parts are built layer by layer. Even if these layers adhere, they may delaminate under specific weights or orientations. While polyjet and multijet parts are frequently brittle, the problem is compounded when goods are manufactured using fused deposition modelling (FDM). In other cases, injection molding may be preferred since it generates homogeneous components that will not split and shatter. Another potential difficulty with 3D printing is the type of equipment or technique used, because certain printers have lower tolerances, which means that the finished result may not match the original design [40]. This can be corrected in post-processing, but keep in mind that it will increase manufacturing time and expense (Table 1).

**Table 1:** Summary of characteristics of included studies PCL, polycaprolactone; ABS, acrylonitrile butadiene styrene; PEEK, polyether ether ketone; FFF, fused filament fabrication

Authors	Year	Findings
Balasundaram et al. [1]	2012	Patients who receive facial allotransplants must be immunosuppressed for the rest of their lives; hence, this form of reconstruction should only be used in rare situations.
Farré-Guasch et al. [2]	2015	Additive manufacturing (AM) is the technique of connecting materials to produce items from digital three-dimensional (3D) model data, and it is a promising technology in oral and maxillofacial surgery.
Kumar et al. [3]	2022	The goal of this study was to compare the outcomes of various reconstructive procedures in 20 individuals who had segmental resection of the mandible between 2004 and 2017.
Alwala et al. [4]	2023	Within the constraints of the study, it is determined that patient-specific implant systems are an effective therapy strategy for the repair of the maxilla afflicted by mucormycosis secondary to Covid-19.
Shilo et al. [5]	2018	Three-dimensional-printed patient-specific implants (PSI) enable accurate reconstruction of anatomical relationships, as well as efficient function restoration.
Dawood et al. [7]	2015	This study examines the various 3D printing (3DP) technologies available and their uses in dentistry and maxillofacial surgery.
Hegedus et al. [8]	2022	3D printing is one of the most important aspects of digital dentistry, and it is predicted to grow rapidly. As a result, dentists must gain relevant information in order to integrate this technology into their daily practice.
Della Bona et al. [9]	2021	A qualitative analysis of papers published on stereolithography (SLA)-based 3D printing of restorative materials and their therapeutic application was conducted in a systematic review.
Louvrier et al. [12]	2017	Dental implant surgery and mandibular reconstruction were the two most common clinical indications. Surgical instructions and anatomical models were the most often printed objects. Forty-five percent of the prints were professional. The primary benefits were increased precision and decreased surgery time.
Zhu et al. [14]	2019	This paper provides a cutting-edge evaluation of new colloidal processing methodologies for the 3D printing of organic, ceramic, metallic, and carbonaceous materials. It is anticipated that concurrent advances in colloid design and 3D printing will open up several opportunities for the manufacture of new constructions not previously possible using existing technologies, considerably broadening their applications.
Kirchmajer et al. [15]	2015	This review assesses hydrogel-forming polymers that are ideal for soft tissue engineering, with a particular emphasis on materials that may be created using additive manufacturing (3D printing). The next section provides an overview of the unique material needed for hydrogel-based tissue engineering constructions.
Sharma et al. [16]	2021	The goal of this study is to explain the selection, manufacture, qualities, and uses of new biological polymer-derived implants such as silk, lignin, soy, collagen, gelatin, chitosan, alginate, and starch.
Arif et al. [18]	2022	This analysis covers present obstacles and future potential in the 3DP of PCL- and polylactic acid (PLA)-based composites that will assist biomedical engineers in meeting clinical demands.
Anadioti et al. [19]	2020	This review discusses current challenges and future opportunities in the 3DP of PCL- and PLA-based composites, which will help biomedical engineers satisfy clinical demands.
Celik et al. [20]	2023	Additive manufacturing (AM), also referred to as 3D printing, has the potential to revolutionize the industry. While there have been advances in the use of AM for dental restorations, more research is needed to develop suitable biomedical and dental materials.
Goodacre and Goodacre [21]	2022	Printing has several advantages over milled and conventionally produced dentures; nonetheless, study is needed to answer many problems. The benefits include lower printer costs when compared to milling equipment, less material waste, the ability to print many dentures at the same time, and the ability to construct intricate shapes that would otherwise be impossible to mill.
Deshmane et al. [24]	2021	Three-dimensional printing (3DP) technology is a cutting-edge tool utilized in the production of medical equipment and alloys, the replacement of biological tissues, the production of customized dosage forms, and other applications. Stereolithography (SLA), a 3D printing technology, is fast and precise, producing completed objects of consistent quality.
Sinn et al. [25]	2006	Computer advancements have improved in the diagnosis and treatment of complex congenital craniofacial abnormalities. In the treatment of craniofacial abnormalities, stereolithographic models are beginning to replace traditional milled models. Stereolithographic models have been found in studies to be highly accurate and to provide additional information in treatment planning for the correction of craniofacial abnormalities.
Chen et al. [26]	2016	A review of the methods is required in template-guided surgery, such as image processing, 3D visualization, preoperative planning, surgical guide creation, and manufacture. Furthermore, template-guided clinical applications for various types of surgeries are evaluated, and it is revealed that surgical precision has improved when compared to non-guided operations.
Matta et al. [27]	2017	In advanced implantology, the use of a surgical template is a well-established procedure. In addition to traditional fabrication, the computer-aided design and computer-aided manufacturing (CAD/CAM) workflow allows for the creation of implant drilling templates using a three-dimensional printer. A highly accurate surgical guide is required to translate the virtual planning to the oral environment.
Melnikova et al. [29]	2018	Selective laser sintering (SLS) and fused deposition modelling (FDM) technology were utilized to 3D-print textile-based structures. Although ABS has been shown to be too fragile for fine structures, hard, polylactic acid (PLA), as well as the nylon used in SLS printing, can be extremely difficult to use in typical textile applications such as garments; soft PLA, also in combination with less elastic materials such as BendLay, has been shown to be capable of reproducing some textile-based structures.
Tan et al. [30]	2016	This study demonstrated that it is possible to create patient-specific acrylic cranioplasty implants using a low-cost 3D printer. More research is needed to determine clinical applicability. This intriguing method has the potential to provide personalized medication to more patients around the world.
Lim et al. [31]	2022	This long-term follow-up study found that patient-specific titanium implants perform well when used to treat various abnormalities in the oral and maxillofacial areas. Instead of autogenous bone, a 3D-printed titanium implant can be used efficiently in the repair of the zygoma and jaw without donor site morbidity.
Mangano et al. [32]	2013	It is possible that treating severely atrophied posterior mandibles with standard-diameter root-form implants will be difficult. Bone reconstructive surgery is the treatment of choice; nevertheless, some patients may refuse it for financial reasons or owing to increased morbidity.
Thimukonda Jegadeesan et al. [33]	2022	Cranioplasty, a century-old treatment that improves structural and functional outcomes, is still used today. Surgical treatments and implant materials have evolved over time and now have a good cosmesis.
Honigmann et al. [34]	2018	Although this paper only covers a tiny portion of the research conducted in the project to construct 3D-printed PEEK PSI utilizing FFF, it does open up a wide range of possibilities for future innovation and advancement in surgical applications.
Suojanen et al. [35]	2016	The utilization of virtual surgery, patient-specific saw and drill guides, and custom-made osteosynthesis plates is fast extending from deformity surgery to orthognathic surgery. The majority of commercially available systems use computer-aided design/computer-aided manufacturing (CAD/CAM) wafers to create patient-specific saw guides.
Moiduddin et al. [37]	2023	Zygoma bone replacement with an implant is one of the most difficult operations in implant dentistry due to the anatomical structure's free-form nature. Because there are so many distinct materials and manufacturing procedures, the three most important factors in cranial reconstruction are implant design, material selection, and fabrication.
Hollister et al. [39]	2016	This paper describes the integration of image-based, multiscale, patient-specific design with 3D biomaterial printing inside a design control framework for clinical translation. We define design inputs for patient-specific implants and scaffolds and then apply image-based patient-specific design to achieve these inputs.

## Conclusions

Surgery planning can benefit financially from 3D printing's low cost. Using CAD technology, a 3D-printed model can be generated in advance of surgery to speed up the process. For the ideal approach and creation of a 3D structure, effective segmentation is carried out. Before surgery, basic knowledge of the procedure was provided by 3D-printed models and implants. The 3D printing technology is widely used, and it is possible to produce intricately designed components. Implants made specifically for each patient are produced using 3D printing technology. In the field of oral and maxillofacial surgery, 3D printing offers a wide range of uses that enable the development of innovative and effective processes for producing client-specific goods.
